# Peritoneal dialysis care during the COVID-19 pandemic, Thailand

**DOI:** 10.2471/BLT.21.286792

**Published:** 2021-11-04

**Authors:** Talerngsak Kanjanabuch, Krit Pongpirul

**Affiliations:** a Division of Nephrology, Department of Medicine and Center of Excellence in Kidney Metabolic Disorders, Faculty of Medicine, Chulalongkorn University, Bangkok, Thailand.; b School of Global Health, Faculty of Medicine, Chulalongkorn University,1873 Rama IV Rd, Patumwan, Bangkok 10330, Thailand.

## Abstract

**Problem:**

The coronavirus disease 2019 (COVID-19) pandemic could affect health service provision of less urgent interventions, such as peritoneal dialysis for chronic kidney disease patients.

**Approach:**

To assess how peritoneal dialysis centres in Thailand adapted their provision of care, we invited medical directors and peritoneal dialysis managers to respond to an online survey on 1 July 2021. We asked whether they had modified or deferred their training, catheter insertion or removal, intravenous supplements, follow-up and home visits, and workload.

**Local setting:**

Patients needing dialysis receive peritoneal dialysis free of charge in Thailand. As of 31 December 2020, 240 peritoneal dialysis centres in Thailand have provided care to 32 284 patients.

**Relevant changes:**

At 24.6% (29/118) of centres, educational sessions for patients were modified. Catheter insertion continued at 71.9% (82/114) of centres. Few facilities (19.7%; 23/117) continued to perform peritoneal equilibration tests as usual. On-site intravenous injections were mostly transferred to health centres close to the patients’ homes. Most centres reduced their outpatient follow-up visits (51.7%; 61/118) and stopped visiting patients at home (66.9%; 79/118). Peritoneal dialysis nurses reported an increased workload at 62.7% (74/118) of centres, and in many instances (66.1%; 78/118) were providing nursing care to COVID-19 patients and administering COVID-19 vaccines.

**Lessons learnt:**

Health-care providers altered clinical care activities to protect their patients from COVID-19. However, further evidence is needed on the consequences of such alteration in care. To prepare for future pandemics, actors need to explore nonconventional peritoneal dialysis care as well as financial and nonfinancial incentive mechanisms for such care.

## Résumé

**Problème:**

La pandémie de maladie à coronavirus 2019 (COVID-19) est susceptible d’affecter la réalisation, par les services de santé, d’interventions moins urgentes, telles que les dialyses péritonéales pour les patients atteints de maladies rénales chroniques.

**Approche:**

Afin d’évaluer comment les centres médicaux procédant à des dialyses péritonéales en Thaïlande ont adapté leur prestation de soins, nous avons invité des directeurs médicaux et des personnes effectuant des dialyses péritonéales à répondre à une enquête en ligne le 1^er^ juillet 2021. Nous leur avons demandé s’ils avaient modifié ou reporté leur formation, l’insertion ou le retrait de cathéters, des suppléments intraveineux, des visites de suivi ou à domicile et leur charge de travail.

**Environnement local:**

En Thaïlande, les patients nécessitant une dialyse bénéficient gratuitement d’une dialyse péritonéale. Au 31 décembre 2020, 240 centres thaïlandais réalisant des dialyses péritonéales ont fourni des prestations à 32 284 patients.

**Changements significatifs:**

Dans 24,6% (29/118) des centres, les séances éducatives destinées aux patients ont été modifiées. L’insertion de cathéters s’est poursuivie dans 71,9% (82/114) des centres. Peu de centres (19,7%; 23/117) ont continué à effectuer le test d’équilibrage péritonéal comme à leur accoutumée. Les injections intraveineuses sur place ont été transférées pour la plupart dans des centres de santé proches du domicile des patients. La plupart des centres ont réduit leurs visites de suivi ambulatoire (51,7%; 61/118) et ont cessé de rendre visite aux patients à leur domicile (66,9%; 79/118). Le personnel infirmier chargé de réaliser des dialyses péritonéales a signalé une augmentation de sa charge de travail dans 62,7% (74/118) des centres et, dans de nombreux cas (66,1%; 78/118), il fournissait des soins infirmiers aux patients atteints de la COVID-19 et vaccinait contre la COVID-19.

**Leçons tirées:**

Les prestataires de soins de santé ont modifié leurs activités cliniques pour protéger leurs patients contre la COVID-19. Cependant, des preuves supplémentaires sont nécessaires sur les conséquences d’une telle modification du schéma de soins. Pour se préparer aux futures pandémies, les acteurs de la santé doivent envisager des interventions de dialyse péritonéale non conventionnelles ainsi que des mécanismes d’incitation financière et non financière pour ces soins.

## Resumen

**Situación:**

La pandemia de la coronavirosis de 2019 (COVID-19) podría afectar a la prestación de los servicios sanitarios de intervenciones menos urgentes, como la diálisis peritoneal para pacientes con enfermedades renales crónicas.

**Enfoque:**

Para evaluar cómo los centros de diálisis peritoneal de Tailandia adaptaron su prestación de cuidados, se invitó a los directores médicos y a los gestores de diálisis peritoneal a responder a una encuesta en línea el 1.^o^ de julio de 2021. Se les preguntó si habían modificado o aplazado su formación, la inserción o retirada de catéteres, los suplementos por vía intravenosa, las visitas de revisión y a domicilio y la carga de trabajo.

**Marco regional:**

Los pacientes que necesitan diálisis reciben diálisis peritoneal de forma gratuita en Tailandia. A 31 de diciembre de 2020, 240 centros de diálisis peritoneal en Tailandia atendieron a 32 284 pacientes.

**Cambios importantes:**

Se modificaron las sesiones educativas para los pacientes en el 24,6 % (29/118) de los centros. La inserción de catéteres continuó en el 71,9 % (82/114) de los centros. En pocos centros (19,7 %; 23/117) se siguió realizando la prueba de equilibrio peritoneal de forma habitual. Las inyecciones por vía intravenosa *in situ* se transfirieron en su mayoría a los centros sanitarios cercanos a los domicilios de los pacientes. La mayoría de los centros disminuyeron sus visitas de revisión ambulatoria (51,7 %; 61/118) y dejaron de visitar a los pacientes en sus domicilios (66,9 %; 79/118). Las enfermeras dedicadas a la diálisis peritoneal informaron de un incremento de la carga de trabajo en el 62,7 % (74/118) de los centros, y en muchos casos (66,1 %; 78/118) estaban proporcionando cuidados de enfermería a los pacientes de la COVID-19 y administrando las vacunas contra la COVID-19.

**Lecciones aprendidas:**

Los proveedores de servicios sanitarios modificaron las actividades de atención clínica para proteger a sus pacientes de la COVID-19. No obstante, se necesitan más evidencias sobre las consecuencias de esta modificación de la atención. Para prepararse para futuras pandemias, los agentes sanitarios deben explorar los cuidados de diálisis peritoneal no convencionales, así como los mecanismos de incentivos financieros y no financieros para estos cuidados.

## ملخص

**المشكلة:**

يمكن أن تؤثر جائحة فيروس كورونا 2019 (كوفيد 19) على تقديم الخدمات الصحية للحالات الأقل إلحاحًا، مثل غسيل الكلى البريتوني لمرضى أمراض الكلى المزمنة.

**الأسلوب:**

من أجل تقييم كيفية قيام مراكز غسيل الكلى البريتوني في تايلند بتكييف تقديمها للرعاية، قمنا بدعوة المديرين الطبيين، ومديري غسيل الكلى البريتوني، للمشاركة في استطلاع عبر الإنترنت في 1 يوليو/تموز 2021. سألنا عما إذا كانوا قد قاموا بتعديل تدريبهم أو تأجيله، أو إدخال القسطرة أو إزالتها، وحقن المكملات عبر الوريد، والمتابعة والزيارات المنزلية، وعبء العمل.

**المواقع:**

**المحلية ** المرضى الذين يحتاجون إلى غسيل الكلى، يتلقون غسيل الكلى البريتوني دون مقابل في تايلند. اعتبارًا من 31 ديسمبر/كانون أول 2020، قدم 240 مركزًا لغسيل الكلى البريتوني في تايلند الرعاية لعدد 32284 مريضًا.

**التغيّرات:**

**ذات** **الصلة ** في 24.6% (29/118) من المراكز، تم تعديل الجلسات التعليمية للمرضى، بينما استمر إدخال القسطرة عند 71.9% (82/114) من المراكز. وحافظ عدد قليل من المرافق (19.7%؛ 23/117) على إجراء اختبار التوازن البريتوني كالمعتاد. تم في الغالب نقل عمليات الحقن في الوريد في الموقع إلى المراكز الصحية القريبة من منازل المرضى. قامت معظم المراكز بتقليل زيارات المتابعة للمرضى الخارجيين (51.7%؛ 61/118)، وتوقفت عن زيارة المرضى في المنازل (66.9%؛ 79/118). أبلغت ممرضات غسيل الكلى البريتوني عن زيادة عبء العمل في 62.7% (74/118) من المراكز، وفي كثير من الحالات (66.1%؛ 78/118) كن يقدمن الرعاية التمريضية لمرضى كوفيد 19، ويقمن بحقن لقاحات كوفيد 19.

**الدروس:**

**المستفادة ** قام مقدمو الرعاية الصحية بتغيير أنشطة الرعاية الإكلينيكية لحماية مرضاهم من كوفيد 19. ومع ذلك، فإن هناك حاجة لمزيد من الأدلة حول النتائج المترتبة على مثل هذا التغيير في الرعاية، وذلك بهدف التحضير للجائحات المستقبلية، كما تحتاج الأطراف الفاعلة إلى استكشاف رعاية غير تقليدية لغسيل الكلى البريتوني، وكذلك آليات للحوافز المالية وغير المالية لمثل هذه الرعاية.

## 摘要

**问题:**

新型冠状病毒肺炎 (COVID-19) 疫情可能影响不太紧迫的干预措施（例如慢性肾病患者的腹膜透析）的卫生服务供给水平。

**方法:**

旨在评估泰国腹膜透析中心如何调整其护理服务，我们邀请了医疗主任和腹膜透析负责人来回答一项于 2021 年 7 月 1 日开展的在线调查。我们询问他们是否调整或延迟了培训、导管置入或移除、静脉注射补充治疗、随访和家访以及其它工作任务。

**当地状况:**

在泰国，需要透析的患者可以接受免费的腹膜透析。截至 2020 年 12 月 31 日，泰国 240 家腹膜透析中心面向 32,284 位患者提供了护理服务。

**相关变化:**

在 24.6% (29/118) 的中心内，面向患者的教育会议进行了调整。71.9% (82/114) 的中心继续提供导管置入服务。少数机构 (19.7%; 23/117) 照常开展腹膜平衡检测。现场静脉注射大多转移到了患者家附近的医疗中心。大多数中心减少了门诊随访 (51.7%; 61/118)，并且停止到患者家中进行访视 (66.9%; 79/118)。腹膜透析护士的报告表明，62.7% (74/118) 的中心工作量增加，在许多情况下 (66.1%; 78/118) 是由于为新型冠状病毒肺炎患者提供护理服务和注射新冠疫苗。

**经验教训:**

医疗卫生机构调整临床护理活动以保护其患者免于感染新型冠状病毒肺炎。但是，需要进一步的证据来说明这种护理调整可能产生的后果。为了今后更好地应对疫情，行动者需要探索非常规的腹膜透析护理以及针对这些护理的财务和非财务的激励机制。

## Резюме

**Проблема:**

Пандемия коронавирусной инфекции 2019 года (COVID-19) может повлиять на оказание медико-санитарной помощи при состояниях, требующих менее срочных вмешательств, таких как перитонеальный диализ у пациентов с хронической болезнью почек.

**Подход:**

Для оценки изменения условий оказания помощи в центрах перитонеального диализа в Таиланде директорам медицинских учреждений и руководителям центров перитонеального диализа было предложено принять участие в электронном опросе мнений, проведенном 1 июля 2021 года. В ходе опроса уточнялось, изменено или отложено их обучение, установка или удаление катетера, добавки к растворам для внутривенного введения, последующее наблюдение и посещения на дому, а также рабочая нагрузка.

**Местные условия:**

Пациенты, нуждающиеся в диализе, получают лечение перитонеальным диализом в Таиланде бесплатно. По состоянию на 31 декабря 2020 года 240 центров перитонеального диализа в Таиланде оказали помощь 32 284 пациентам.

**Осуществленные перемены:**

В 24,6% (29/118) центров образовательные занятия для пациентов были изменены. Установка катетеров продолжалась в 71,9% (82/114) центров. В немногих учреждениях (19,7%; 23/117) тест перитонеального равновесия проводился в обычном режиме. Проведение процедур внутривенного введения лекарственных средств на месте в основном переносилось в медицинские центры, расположенные недалеко от домов пациентов. Большинство центров сократили число контрольных посещений (51,7%; 61/118) и перестали посещать пациентов на дому (66,9%; 79/118). Медсестры, проводящие перитонеальный диализ, сообщили о возросшей нагрузке в 62,7% (74/118) центров, и во многих случаях (66,1%; 78/118) они обеспечивали сестринский уход за пациентами с COVID-19 и вводили вакцины против COVID-19.

**Выводы:**

Поставщики медицинских услуг изменили виды медицинской помощи для защиты пациентов от COVID-19. Однако необходимы дополнительные данные о последствиях такого изменения ухода. Для подготовки к будущим пандемиям участникам необходимо изучить нетрадиционные методы лечения перитонеальным диализом, а также механизмы финансового и нефинансового стимулирования такого лечения.

## Introduction

The coronavirus disease (COVID-19) pandemic has challenged health systems worldwide. In Thailand, the pandemic has gradually affected the health-care provision for other diseases than COVID-19, including peritoneal dialysis for patients with chronic kidney diseases. These patients, as other people living with comorbidities, are more likely to experience more severe COVID-19 and die of the disease.^1^

As of 30 September 2021, 379 peritoneal dialysis patients had been infected with COVID-19 in the country. Of these, 118 died in the hospital or within 3 months after the onset of infection, 144 recovered, and 117 were still hospitalized (the Peritoneal Dialysis Advisory Board, Nephrology Society of Thailand, unpublished data, 1 October 2021). Furthermore, the massive influx of COVID-19 patients to intensive care units increased the risk of critically ill peritoneal dialysis patients being denied intensive treatment, despite the fact that most dialysis patients had a chance of survival when treated properly. Decreasing living organ donation and a decline in deceased transplant donation also increased the risk of dying for patients waiting for a kidney transplant.

As a nonurgent intervention, peritoneal dialysis services for people with kidney disease were also affected by the overwhelmed health system. Here we describe how peritoneal dialysis centres in Thailand adapted their services to protect their patients during the COVID-19 pandemic.

## Local setting

Thailand’s universal coverage scheme provides financial support for selected health-care services to the majority of Thai citizens. In 2008, the Peritoneal Dialysis First policy was added to the scheme to ensure that patients with chronic kidney diseases have access to free-of-charge dialysis care. Continuous ambulatory peritoneal dialysis is provided as the first-line dialysis modality, whereas the patients with peritoneal dialysis contraindication or a history of technical failure associated with peritoneal dialysis can receive haemodialysis without additional payment. Patients who are medically suitable for peritoneal dialysis can choose haemodialysis as the first-line treatment, but they have to fully self-fund dialysis-related costs.^2^^,^^3^ Medications, including short-acting erythropoiesis-stimulating agents, as well as 2-litre manual glucose-based dialysates (capped at five bags daily), are fully reimbursed if they are listed in the Thai National List of Essential Medicine. 

The policy has triggered an increase in the number of peritoneal dialysis patients and dialysis centres. In 2016, the prevalence and incidence of patients needing peritoneal dialysis were 26 450 (395 per million population) and 10 783 (161 per million population), respectively.^4^ The proportion of peritoneal dialysis patients among total dialysis population has increased from 5.5% (1198/21 839) before 2008 to 30.7% (26 450/86 116) in 2016.^4^

As of 31 December 2020, there were 3 2284 peritoneal dialysis patients, 1176 nephrologists, 637 peritoneal dialysis nurses and 240 peritoneal dialysis centres in Thailand. These centres are usually located in a designated area of a hospital, separated from the outpatient department, and are run by a peritoneal dialysis nurse under supervision of a nephrologist. Most facilities make nurse-led home visits, nurses from 54.5% (12/22) of the facilities visit all patients, while nurses from 45.5% (10/22) of facilities visit selected patients.^5^

## Approach

To assess how peritoneal dialysis centres adapted to the changed circumstances, on 1 July 2021 we invited medical directors and peritoneal dialysis managers from centres across Thailand to participate in an one-time online survey about service provision during the pandemic. If two representatives from the same centre provided conflicting responses to the survey, we communicated with both to solve the conflict. 

Under normal circumstances, new patients receive both individual and group trainings on-site. However, during the outbreak, centres could reduce the number of training days and the hours of training per day. The on-site training and group activities could be replaced by virtual or already available video-assisted training modules. 

For implantation or replacement of the catheter, some clinics only performed these procedures when deemed clinically necessary, if a patient required urgent kidney replacement therapy, or if catheter-related infection or malfunction occurred. Other centres postponed the catheter placement despite prolonging the uraemic condition and lowering quality of life for the patient. The semiquantitative peritoneal equilibration test, which is used to assess peritoneal membrane transport function, could be stopped or only performed when clinically necessary.

To further reduce in-person interactions, centres could either stop providing intravenous iron and nutritional supplements or injection of erythropoiesis-stimulating agent on-site, switch to oral supplements or direct these services to health-care facilities for patients uncomfortable with self-administration. 

The centre could also increase the interval of the regular outpatient follow-up visits or change to a hybrid between in-person and virtual follow-up. The outpatient centre implemented essential measures, such as physical distancing and minimizing contact time. In-centre peritoneal dialysis catheter exit-site dressing was done only for patients with complications. Home visits by a peritoneal dialysis nurse could either be stopped or only performed for patients with post-peritonitis.

## Relevant changes

A cohort of 22 peritoneal dialysis centres showed that, compared with 2019, the number of catheter insertions significantly reduced during 2020 (Table 1).^6^^,^^7^ Catheter removal and re-insertion remained relatively unchanged because delaying such intervention might harm patients and increase mortality.^8^ Hospitalization of patients significantly reduced by 10% (from 895 hospitalizations to 805), presumably due to the reallocation of beds to COVID-19 patients (Table 1).

**Table 1:** Characteristics of peritoneal dialysis care, Thailand, 2019–2020

Variable	No. (%)		*P* ^b^
	2019 (*n* = 2482)^a^	2020 (*n* = 3496)^a^	
New cases	1281 (51.6)	1027 (29.4)	< 0.001
Catheter insertion	1271 (51.2)	1027 (29.4)	< 0.001
Catheter re-insertion	7 (0.3)	6 (0.2)	0.37
Catheter removal	42 (1.7)	41 (1.2)	0.09
Hospitalization	895 (36.1)	805 (23.0)	< 0.001
Death	124 (5.0)	137 (3.9)	0.05

^a^ Active cases.

^b^ We used *χ^2^* test.

Note: The data is from the Peritoneal Dialysis Outcomes and Practice Patterns Study, which have surveyed 22 peritoneal dialysis facilities, each of which provide treatment to at least 20 peritoneal dialysis patients.^6^^,^^7^

In total 198 professionals responded to our survey, covering 118 centres. The survey revealed that the peritoneal dialysis centres took different actions to protect their patients from acquiring COVID-19, and many of the actions were to reduce the person-to-person interactions. For example, 75.4% (89/118) of centres attempted to provide a full educational session to their new patients, whereas the remaining quarter either reduced the training duration or changed to video-assisted training or e-learning technology. 

The majority of centres continued to do catheter insertion (71.9%; 82/114), while 24.6% (28/114) only performed the procedure when clinically necessary. Few centres (19.7%; 23/117) continued to perform peritoneal equilibration tests as usual; most centres (55.6%; 65/117) only performed this procedure in selected patients, including patients with ultrafiltration failure or inadequate dialysis. On-site intravenous injections were mostly transferred to health centres close to the patients’ homes. Only 32.2% (38/118) of the centres continued the regular outpatient follow-up visits, whereas 51.7% (61/118) increased the follow-up interval and 16.1% (19/118) switched to video conferencing, text messaging or telephone calls (Table 2). The low uptake of virtual follow-up could be explained by the fact that 71.8% (84/117) of the centres did not have full teleconferencing facilities for provider–patient communication.

**Table 2:** Changes in service provision of peritoneal dialysis centres during the COVID-19 outbreak, Thailand, Jul–Aug 2021

Service	No. (%)			
	Responding centres (*n* = 240)^a^	No change	Deferred	Modified
**Patient training **	118 (49.2)	89 (75.4)	1 (0.8)	28 (23.7)^b^
**Catheter insertion**	114 (47.5)	82 (71.9)	4 (3.5)	28 (24.6)^c^
**Catheter removal**	118 (49.2)	98 (83.1)	7 (5.9)	13 (11.0)^d^
**Visit**				
Outpatient	118 (49.2)	38 (32.2)	61 (51.7)	19 (16.1)^b^
Home	118 (49.2)	18 (15.3)	79 (66.9)	21 (17.8)^e^
**Peritoneal equilibration test**	117 (48.8)	23 (19.7)	29 (24.8)	65 (55.6)^f^
**Intravenous injection**				
Iron supplements	115 (47.9)	38 (33.0)^f^	8 (7.0)^f^	39 (33.9)^g^
Nutritional supplements	115 (47.9)	13 (11.3)^g^	12 (10.4)^g^	7 (6.1)^h^
Erythropoiesis-stimulating agent	118 (49.2)	19 (16.1)	0 (0.0)	99 (83.9)^d^

COVID-19: coronavirus disease 2019.

^a^ No. of peritoneal dialysis centres in Thailand.

^b^ Modified practices were either shortening the duration, increasing the interval or switching to telemedicine.

^c^ Catheter insertion was performed only when clinically necessary.

^d^ Transferred to health centres close to the patients’ home.

^e^ Only visited post-peritonitis patients.

^f^ Only performed when clinically necessary.

^g^ Only 84 facilities provided iron supplement injections to their patients before the pandemic.

^h^ Only 32 facilities provided nutritional supplement injections to their patients before the pandemic.

Notes: Inconsistencies in some values may arise due to rounding. We obtained the data from the Continuous Ambulatory Peritoneal Dialysis Centre Activity Survey.

Home visits made by a peritoneal dialysis nurse were stopped by 66.9% (79/118) of centres, while 15.3% (18/118) of centres still visited their patients and 17.8% (21/118) of centres only visited patients with post-peritonitis (Table 2).

More than half of the centres (62.7%; 74/118) reported an increased workload for the peritoneal dialysis nurses, whereas 4.2% (5/118) reported the opposite. More than half (66.1%; 78/118) of the nurses had to perform nonperitoneal dialysis functions, such as providing nursing care to COVID-19 patients and administering COVID-19 vaccines. The majority of centres (94.9%; 112/118) did not encounter a problem with shipment of dialysis bags during the pandemic.

## Lessons learnt

Besides increased risk of severe COVID-19, patients on peritoneal dialysis in Thailand have experienced changes in practices and procedures linked to their care during the pandemic (Box 1). To substitute outpatient and home visits, some centres attempted telemedicine but the efficacy of this approach is unclear because of the poor patient compliance, poor internet access – especially on the patient side – and unclear financial incentives to the health-care providers. Further research is needed to investigate if telemedicine is a suitable option for patients on peritoneal dialysis, since an effective physical assessment must be done in person. Pitting oedema, cuff and tunnel infections require a physical examination, and cloudy dialysate fluid needs a close-up visual inspection. However, several precise monitoring solutions are available, such as digital weight recording devices and remote patient monitoring devices with pulse oximeters to assess fluid retention. Financing mechanisms for nonconventional peritoneal dialysis care through telemedicine should be further explored.

Box 1 **Summary of main lessons learnt**

- An infectious disease outbreak could reduce the number of nonurgent interventions, such as catheter insertion for peritoneal dialysis.
- The efficacy of using telemedicine as a substitute for outpatient and home visits is unclear due to poor patient compliance, poor internet access and unclear financial incentives to the health-care providers.
- While health-care providers have been trying to reduce or modify medical care activities as they see appropriate, further evidence is needed on the consequences of such alterations in care.

As the first two COVID-19 waves only affected three and one peritoneal dialysis patients, respectively, experts hypothesized that many centres were not fully prepared for the large wave starting on 23 April 2021, although recommendations on care for noninfected peritoneal dialysis patients were published in May 2020.^9^ Nevertheless, to protect their patients from acquiring COVID-19, health-care providers at most centres reduced or modified medical-care activities as they saw appropriate. However, some of these approaches might have negatively affected the clinical care process for some patients, and the patients might not fully understand how the changes in care would affect them. Hence, further evidence is needed on the consequences of nonconventional disease-specific care. Changes in care also had to be supported by the already fixed global budget, and key performance indicators^10^ could be adversely affected by the COVID pandemic. 

Peritoneal dialysis patients infected with COVID-19 faced additional challenges since the government had mandated that patients had to be admitted to the hospital that made the diagnosis. Some of these hospitals are unable to provide peritoneal dialysis care, and even if patients were admitted to a peritoneal dialysis-equipped facility, many patients had to transfer from manual peritoneal dialysis to either automated peritoneal dialysis or haemodialysis. This transfer might put the patients at risk of retaining salt and middle-molecule toxins, despite equivalence in the effectiveness of manual and automated modalities being demonstrated in meta-analysis studies.^11^^,^^12^

To prepare for future pandemics, peritoneal dialysis centres need to explore innovative approaches for education as well as physical and laboratory assessments of patients. Furthermore, the public health insurance schemes should introduce financial and nonfinancial incentive mechanisms for nonconventional peritoneal dialysis care, including telemedicine activities, and relevant professional associations should develop guidelines for the care of patients with chronic diseases during infectious outbreaks.
